# A box of chocolates

**DOI:** 10.1212/NXI.0000000000000234

**Published:** 2016-06-02

**Authors:** Josep Dalmau

**Affiliations:** From IDIBAPS, Service of Neurology, Hospital Clínic, Barcelona, Spain.

The articles in this issue of *Neurology® Neuroimmunology & Neuroinflammation* cover a broad range of topics, including new insights into the pathogenesis of natalizumab-associated progressive multifocal leukoencephalopathy (PML), important observations on antibody testing in neuromyelitis optica spectrum disorders (NMOSD), and a study related to degenerative dementia. However, all the articles are of interest. Like the famous box of chocolates anecdote from the movie *Forrest Gump*, you never know what you are going to get, but you know it will be enjoyable.

PML is a serious complication of natalizumab. Risk factors in patients with multiple sclerosis (MS) are seropositivity for JC virus, prior treatment with immunosuppressants, and duration of natalizumab treatment, with the highest incidence occurring after 24 monthly doses.^1^ However, patients with MS with no natalizumab treatment and patients with other disorders with similar histories of immunosuppression and JV virus seropositivity have much lower PML risks, suggesting something specific to natalizumab effects on downstream pathways that results in JC viral activation and PML. Therefore, the study by Meira et al.^2^ elucidating a pathway by which natalizumab may promote PML is exciting. These authors previously found that the transcription factors POU2AF1 and Spi-B were upregulated in CD4+ T cells after 24 months of natalizumab dosing but not after shorter dosing periods.^3^ POU2AF1 is a critical regulator of Spi-B that binds to unique sequences of the JC virus and drives viral activity. The current study extends these findings and demonstrates that B and CD8+ T cells from patients treated with natalizumab, but not from natalizumab-naive patients, also show upregulation of these factors. In these cells the upregulation occurs early and increases over time with maximal effect seen after 24 months. These findings demonstrate that natalizumab produces a sustained pattern of altered gene expression that is dependent on treatment duration and most pronounced after 2 years. In an accompanying editorial, Major and Nath^4^ incorporate these data into an elegant proposal, linking natalizumab with the JC viral pathogenesis of PML. This will surely be an active area of research that we will continue to report on.

In 2015, the International Panel for NMO Diagnosis proposed new diagnostic criteria that dispensed with the term neuromyelitis optica (NMO) in favor of unifying patients under the term NMOSD.^5^ The criteria were based on the presence or absence of aquaporin-4 immunoglobulin (AQP4-IgG) and on the assumption that patients with NMO/limited forms would develop a typical NMO syndrome over time. Is this correct and does antibody status trump clinical phenotype for predicting outcome? Answers are provided by Sepulveda and colleagues,^6^ who carried out a comparative study of a large cohort of patients who fulfilled the 2006 NMO criteria or were diagnosed with NMO/limited forms with AQP4-IgG. With a median follow-up of over 6 years, the authors found that all AQP4-IgG-seropositive patients had similar disability outcomes, including those who remained with a limited form phenotype. Among the AQP4-IgG-seronegative patients, almost a third of the cases were found to have myelin oligodendrocyte glycoprotein IgG (MOG-IgG). The double-seronegative patients had similar outcomes to patients with AQP4-IgG, whereas the patients with MOG-IgG had better disability outcomes. These data support the use of the term NMOSD for AQP4-IgG-seropositive patients and suggest that MOG-IgG testing be carried out in AQP4-IgG-seronegative patients, a point not considered in the new NMOSD criteria.^5^

The importance of AQP4-IgG serostatus in the 2015 NMOSD diagnostic criteria makes the study of Majed et al.^7^ timely. This study investigates the type of specimen, serum or CSF, and testing method that are more sensitive and specific for AQP4-IgG. The authors studied time-matched serum and CSF samples (defined as being drawn no more than 30 days apart) from cases classified as NMO by 2006 Wingerchuk criteria.^8^ Each sample and a variety of controls were tested with a commercially available M1-AQP4-transfected, fixed cell-based assay (CBA), and in-house live cell flow cytometry. For both methods, the results showed that serum was more informative than CSF for detecting AQP4-IgG and that testing CSF does not provide any additional benefit if serum testing is negative. In carrying out this study, the authors made an interesting observation. The frequency of CSF submission as the sole initial specimen was 1 in 50 in 2007 and 1 in 5 in 2015. They attribute this shift to increased awareness of anti-NMDA receptor encephalitis, in which CSF has been established as the more sensitive and specific specimen. This study is an excellent reminder of the importance of elucidating optimal testing procedures and applying to clinical practice.

Similar to the importance of the type of sample and methodology for antibody testing, there are many variables that need to be accounted for when interpreting neuroimaging studies. This is nicely shown by Finke et al.,^9^ who assessed volume and microstructural integrity of deep gray matter structures in a homogeneous cohort of patients with NMOSD. All patients were Caucasian with AQP4-IgG, almost half had a classic NMO phenotype, and all but 1 had been treated with immunosuppressive therapy. Using 2 different methodologic approaches to measure deep gray matter volume, and diffusion tensor imaging for microstructural integrity, the results demonstrated no differences between the patients with NMOSD and healthy controls matched for age, sex, and years of education. The authors discuss previous studies with inconsistent findings, pointing out that this is likely due to heterogeneity of the study populations without adequate control for factors such as age, sex, or educational background that impact gray matter volume.

The study by Knight et al.^10^ moves us to the realm of degenerative dementias. IV immunoglobulin (IVIg) has been considered attractive as a treatment for Alzheimer disease because it contains anti-amyloid (Aβ) antibodies and has immunomodulatory and anti-inflammatory properties that are emerging as relevant to Alzheimer pathogenesis and progression.^10^ However, despite intriguing preclinical results and positive cognitive effects within subgroups of patients in a phase III clinical trial, IVIg has not been successful in altering Alzheimer disease course. Knight et al.^10^ aimed to link specific anti-Aβ antibodies present in IVIg to clinical activity. The authors used transgenic mice that develop memory deficits and transgene-specific accumulations of Aβ assemblies. In these mice, IVIg prevented the development of memory deficits in specific tasks and this was associated with a reduction in 2 subtypes of soluble-prefibrillar oligomeric Aβ assemblies in the brain. IVIg depleted of antibodies against certain conformers of soluble Aβ assemblies abrogated its therapeutic effect in preserving memory. These data link specific anti-Aβ antibodies with biologic effects and this is an important step in developing potentially more efficacious disease-specific IVIg formulations, providing further evidence that soluble Aβ assemblies may play a role in early-stage Alzheimer processes.

In keeping with my chocolate theme, one could say that case reports are like bonbons—small but packed full of interesting flavor. The current issue includes a patient with IgM neuropathy associated with Waldenström macroglobulinema successfully treated with rituximab (Kadoya et al.),^11^ a pregnant patient with Kikuchi-Fujimoto disease who developed NMOSD (Kaku et al.),^12^ and a patient with VGCC-associated cerebellar degeneration and Lambert-Eaton myasthenic syndrome (McKasson et al.).^13^ There are additional, equally interesting articles in this issue and I hope you will enjoy them all.
